# Chronic Insulin Infusion Down-Regulates Circulating and Urinary Nitric Oxide (NO) Levels Despite Molecular Changes in the Kidney Predicting Greater Endothelial NO Synthase Activity in Mice

**DOI:** 10.3390/ijms19102880

**Published:** 2018-09-22

**Authors:** Maurice B. Fluitt, Sophia Rizvi, Lijun Li, Ashley Alunan, Hwal Lee, Swasti Tiwari, Carolyn M. Ecelbarger

**Affiliations:** 1 Division of Endocrinology and Metabolism, Department of Medicine, Georgetown University, Washington, DC 20057, USA; mbf79@georgetown.edu (M.B.F.); sophiarizvi@health.usf.edu (S.R.); lil@georgetown.edu (L.L.); amalunan@willamette.edu (A.A.); hwal.lee17@gmail.com (H.L.); 2 Willamette University, Salem, OR 97301, USA; 3 Department of Molecular Medicine & Biotechnology, Sanjay Gandhi Postgraduate Institute of Medical Sciences, Lucknow 226014, India; tiwari_pgi@yahoo.com

**Keywords:** heart rate, blood pressure, oxidative stress, metabolic syndrome, hypertension

## Abstract

Insulin therapy is often needed to overcome insulin receptor resistance in type 2 diabetes; however, the impact of providing additional insulin to already hyperinsulinemic subjects is not clear. We infused male TALLYHO/Jng (TH) mice (insulin resistant) with insulin (50 U/kg·bw/d) or vehicle (control) by osmotic minipump for 14 days. One group of insulin-infused mice was switched to 4% NaCl diet (high-sodium diet, HSD) in the second week. Blood chemistry revealed a significantly higher anion gap and blood sodium concentrations with insulin infusion, i.e., relative metabolic acidosis. Systolic BP and heart rate were slightly (~5 mm Hg) higher in insulin-infused versus control mice. HSD resulted in a modest and transient rise in mean arterial blood pressure (BP), relative to control or insulin-infused, normal-NaCl-fed mice. In kidney, insulin infusion: (1) increased total and phosphorylated (serine-1177) endothelial nitric oxide synthase (eNOS) band densities; (2) reduced band density of the uncoupled form of eNOS; and (3) increased renal homogenate nitric oxide synthase (NOS) activity. Despite this, plasma and urine levels of nitrates plus nitrites (NOx) fell with insulin infusion, by day 14 (40–50%) suggesting worsening of resistance. Overall, insulin infusion ramps up the cellular means in kidney to increase vasodilatory and natriuretic NO, but in the long term may be associated with worsening of insulin receptor resistance.

## 1. Introduction

Insulin therapy is often used to treat diabetic subjects quite successfully and lower blood glucose; however, the cardiovascular impact of additional circulating insulin is not certain. Insulin infusion (to produce hyperinsulinemia) has been shown to have conflicting effects on blood pressure (BP) depending on strain and species studied. Hall and associates demonstrated increased blood pressure (BP) in rats [1,2], but not dogs [3], infused with insulin. We showed increased BP in Sprague-Dawley rats infused with insulin for 30 days [4], but not in C57Bl6 mice [5]. Knockout of the insulin receptor using a Cre-lox recombinase approach from portions of the renal tubule by our group has resulted in different BP phenotypes depending on whether we targeted the whole-renal tubule [6] or just the collecting duct principal cell [7]. Many of the augmentative effects of insulin on BP have been related to insulin’s anti-natriuretic actions and its ability to directly activate many different sodium transport and channel activities along the renal tubule [8,9,10,11].

In contrast, insulin may also reduce BP by its ability to regulate NO production via activation of Akt (protein kinase B) and phosphorylation and activation of nitric oxide synthases (NOS). A net increase in NO bioavailability is associated with vasodilation and natriuresis in response to signaling through cyclic GMP, both which would tend to lower BP. Endothelial NOS (eNOS or NOS3), which is found not only in endothelial cells, but also along the renal tubule epithelia [12], has been shown to be phosphorylated at serine 1177 by insulin, thereby increasing its activity [13]. However, insulin has also been shown to increase phosphorylation of eNOS on tyrosine residue 657, a modification associated with reduced eNOS activity [14]. In addition, eNOS activity can become “uncoupled” meaning that it switches from an efficient generator of solely NO and l-citrulline from l-arginine and O_2_ to a partial generator of O_2_^•−^ (superoxide) [15]. This apparently occurs due to slowed or inefficient transport of electrons to the ferrous-heme-O_2_ intermediate resulting in generation of free radicals, such as peroxynitrite. Thus, the net effect of insulin on NO generation in vivo may be situational, and the absolute effect of insulin, in the absence of receptor resistance, on BP and other hemodynamic factors, is uncertain.

Moreover, insulin may also be hypertension-promoting, due to the development of insulin receptor (Insr) resistance. Insulin resistance refers to reduced activation of canonical Insr signaling cascades, which involve initially phosphorylation of the receptor itself following by activation of phosphoinositide-3-kinase (PI-3K) signaling. Elevated circulating insulin may also have the potential to bind and activate insulin-like-growth factor receptors (IGF), which also may promote sodium retention [8,9]. Insulin resistance is often associated with oxidative stress and an elevation in the renin-angiotensin system [10]. Reduced Insr signaling efficiency is first-and-foremost associated with impaired glucose clearance from the blood, i.e., glucose intolerance, but may lead to a number of other abnormalities, as insulin is involved in the reabsorption of other substances in the kidney and in nitric oxide (NO) production in the kidney and vasculature [11].

The aim of these studies was to characterize the physiologic response of mice with pre-existing insulin resistance to infused insulin with a focus on the factors related to BP control. We infused TALLYHO/Jng (TH) mice with insulin (50 U/kg·bw/d) for two weeks. TH mice were developed by Jackson Laboratory by selectively inbreeding mice of the Theiler Original colony for hyperglycemia [16]. Several multiple quantitative trait loci have been identified in these mice that are linked to adiposity, hyperglycemia, insulin resistance, and type 2 diabetes [16]. Although female TH mice are obese, they are much more resistant to developing type 2 diabetes; thus we chose to study only males in this particular study. We focused on the kidney, as several studies suggest that the kidney does not develop Insr resistance to the same extent as tissues, such as liver, adipose, and muscle [17,18]. This has been provided as a reason for why hyperinsulinemia may raise BP (due to inappropriate sodium reabsorption in the kidney) [17]. We examined BP responses by radiotelemetry, as well as, changes in blood chemistry, and the expression, phosphorylation, and activity of eNOS, and its substrate competing enzyme, arginase.

## 2. Results

### 2.1. Blood Pressure

**Figure 1:**
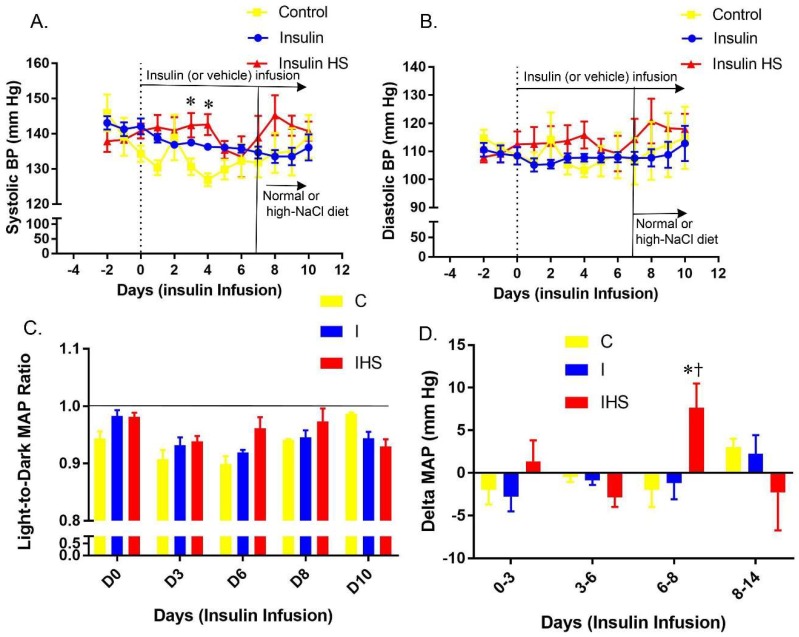
Blood pressure—Daily mean (**A**) systolic and (**B**) diastolic blood pressures in control (vehicle infused, 1% NaCl diet), insulin infused (fed 1% NaCl all study) and insulin infused plus high-NaCl diet (4%, high NaCl second week,) treated mice; (**C**) light-to-dark period mean arterial pressure (MAP) ratio on various days; (**D**) delta (change) in mean arterial pressure (MAP) in various periods; (mean ± sem, *n* = 4–7/group); * indicates a significant difference from control and † from insulin-treated, by multiple comparisons testing following a significant one-way analysis of variance (ANOVA, *p* < 0.05).

The systolic and diastolic blood pressures (BP) for the mice are shown in Figure 1A,B, respectively. In the first week, all mice were fed a normal salt (1% NaCl) diet. Insulin infusion did not markedly affect diastolic or systolic BP in the first week. The addition of a high-sodium diet (HSD), (red line/symbols) caused a 5–7 mm increase in both systolic and diastolic BP, suggesting marginal salt-sensitivity. Figure 1C shows the light-to-dark ratio of mean arterial pressure (MAP) on various days to evaluate the effect of treatments on diurnal rhythm of BP. Over the course of the experiment, ratios were below 1.0 for all groups, indicating MAP fell during the light period, as expected. No major effects of treatment were found. The change in mean arterial pressure MAP, in various periods, is shown in Figure 1D. IHS-treated (Insulin, high-NaCl diet) mice had a significantly greater rise in MAP between days 6–8 (period when the HSD was initiated), as compared to I (Insulin, normal NaCl diet) and C (vehicle, normal NaCl diet) groups.

### 2.2. Heart Rate

**Figure 2:**
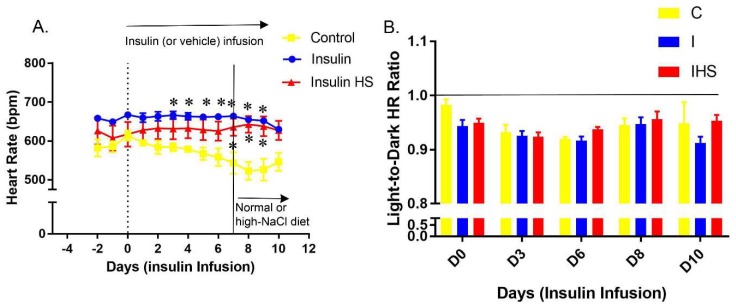
Heart rate—(**A**) Daily average heart rate (HR) in control (vehicle infused, 1% NaCl diet), insulin infused (fed 1% NaCl all study) and insulin infused plus high-NaCl diet (4%, HS) treated mice; in various periods (**B**) light-to-dark period HR ratio on various days (mean ± sem, *n* = 4–7/group); * indicates a significant difference from control, by multiple comparisons testing following a significant one-way ANOVA (*p* < 0.05).

The mean heart rates (HR) of the mice are shown in Figure 2A. HR was fairly stable early, then fell by about 50 beats/min in the second week with insulin infusion. Figure 2B shows the light-to-dark ratio of HR. Light-to-dark ratio decreased slightly in the beginning of the insulin infusion, but normalized by the end of the study. Light-to-dark ratio of HR was not sensitive to HSD.

### 2.3. Physiological Parameters and Blood Chemistry

**Figure 3:**
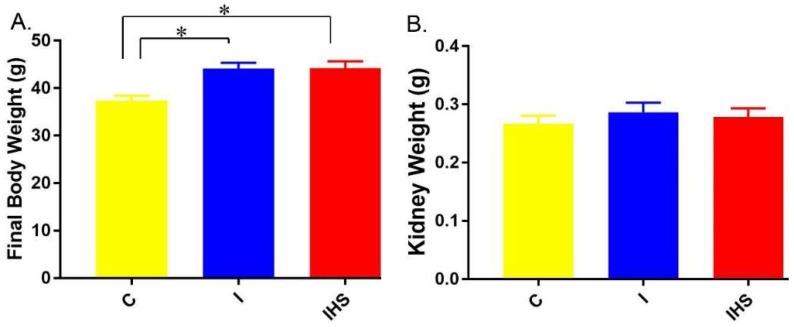
Body and kidney weights—(**A**) Final mean body weights in the three groups; (**B**) final kidney weights (average right and left kidneys); * indicates a significant (*p* < 0.05) difference between groups (mean ± sem, *n* = 11–12/group).

Final body and kidney weights of mice are shown in Figure 3. Insulin infusion increased body weight and there was no impact of the HSD in the second week. Kidney weights were not affected by treatment.

Blood chemistry is shown in Table 1. Plasma insulin concentrations were increased about four-fold with infusion. Blood sodium (Na^+^) concentrations along with the anion gap (AnGap) were significantly increased by insulin infusion, and then normalized some by the addition of the HSD. Similarly, blood pH decreased significantly with insulin infusion, and this decrease was attenuated by HSD. Blood chloride (Cl^−^) was significantly decreased by insulin infusion in combination with the HSD. Blood urea nitrogen (BUN) was markedly reduced by insulin infusion versus Control mice. Blood potassium (K^+^), total CO_2_, glucose, hematocrit, bicarbonate (HCO_3_^−^), base excess, and hemoglobin levels were not significantly affected by treatment.

### 2.4. Plasma Insulin, NOx, and Arginase Activity

**Figure 4:**
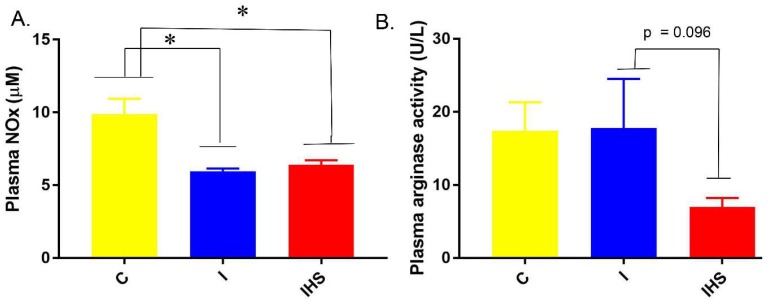
Plasma parameters—(**A**) Insulin concentration; (**B**) nitrates plus nitrites (NOx) concentration; * indicates a significant (*p* < 0.05) difference between groups (mean ± sem, *n* = 7–8/group).

Insulin infusion reduced circulating levels of plasma nitrates plus nitrites (NOx) concentrations, similarly with no effect of the HSD (Figure 4A). Plasma arginase (competitor for arginine with nitric oxide synthase) activity was not significantly different, due to treatment, but trended toward being reduced by HSD (*p* = 0.096 as compared to insulin alone, Tukey’s multiple comparisons test, Figure 4B).

### 2.5. Urine and Kidney NO Regulation

**Figure 5:**
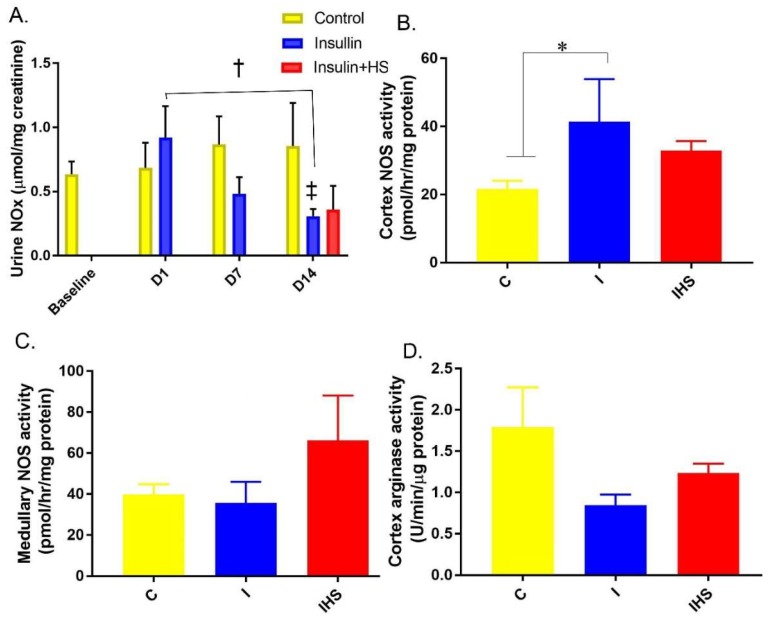
Urine NOx activity—(**A**) Urine NOx (24-h collection) during baseline and days 1, 7, and 14; (**B**) cortex NOS activity; (**C**) Medulla NOS activity (combined inner medulla and inner stripe of the outer medulla); (**D**) cortex arginase activity; (mean ± sem, *n* = 8–12/group for (**A**–**C**) and 3–4/group for (**D**)). * indicates a significant difference between groups (*p* < 0.05); ‡ indicates a significant difference from baseline, and † between identified days, by two-way repeated measures (RM) ANOVA followed by multiple comparison’s testing.

Urinary NOx (measured at several time-points) was gradually reduced with both chronic insulin infusion and insulin plus HSD (Figure 5A). Cortical NOS activity was increased by insulin infusion, a rise which was attenuated by HSD (Figure 5B). Medullary NOS activity, in contrast, was not significantly affected by treatment (*p* = 0.26, one-way ANOVA). Cortex arginase activity, similarly, was not significantly affected by treatment (*p* = 0.34, one-way ANOVA).

### 2.6. Kidney eNOS Protein

**Figure 6:**
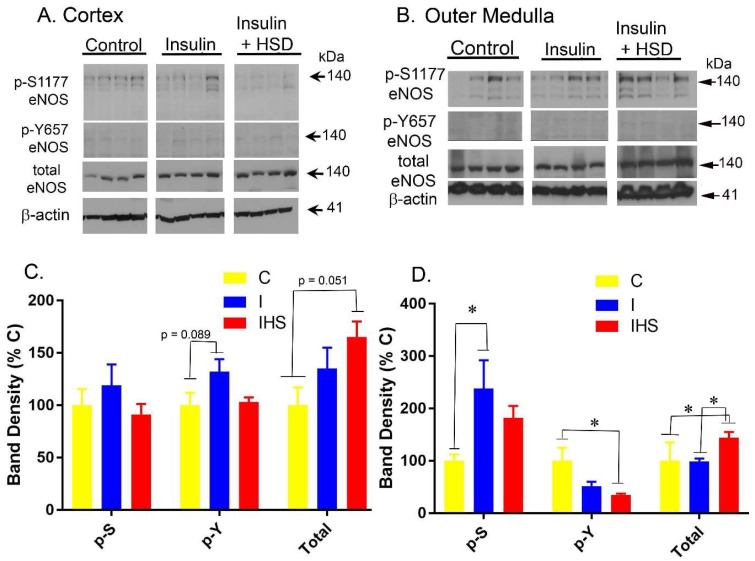
Western blotting for endothelial nitric oxide synthase (eNOS) protein—(**A**) Representative Western blots of cortex homogenates. Separate blots were probed for p-S1177-eNOS, p-Y657-eNOS, and total eNOS. Equal amounts of protein were loaded in each lane. Summaries of band densities (normalized to β-actin) for (**B**) p-S1177-eNOS, p-Y657-eNOS, total eNOS; (**C**) Representative western blots of outer medulla homogenates. Summaries of band densities (normalized to β-actin) for (**D**) p-S1177-eNOS, p-Y657-eNOS, total eNOS; ∗ indicates a significant difference between groups by one-way ANOVA (mean ± sem; *n* = 8–12/group for statistics).

Representative Western blotting for pS1177-eNOS (activating phospho-site), pY657-eNOS (attenuating phospho-site), and “total” eNOS (using a non-phospho-site discriminating antibody) in cortex and inner stripe of the outer medulla homogenates is shown in Figure 6. PS1177-eNOS was increased in the outer medulla by insulin. PY657-eNOS, on the other hand, was reduced in the IHS group in outer medulla. Total band density for eNOS (using the non-phospho-discriminating antibody) showed an increase in the IHS group in outer medulla, with a strong trend (*p* = 0.061) for cortex.

**Figure 7:**
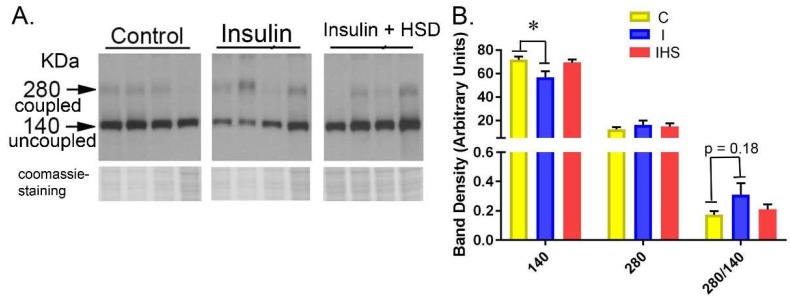
Western blot of eNOS from unreduced samples—(**A**) Representative Western blot for eNOS run on cortex homogenates solubilized with Laemmli without dithiotrietol (DTT) and run under non-reducing conditions. Arrows indicate coupled (active, 280 kDa) and uncoupled (140 kDa) bands. (**B**) Band densities summary; * indicates a significant difference between groups by one-way ANOVA (*p* < 0.05, mean ± sem; *n* = 8/group for statistics).

To assess the uncoupling of eNOS, Western blots were prepared on cortex homogenate samples solubilized and electrophoresed under non-reducing conditions (Figure 7). The uncoupled (inactive) form of eNOS runs at approximately 140 kDa, and the coupled (active) form at 280 kDa. We found a reduction in the uncoupled form of eNOS (140 band) with insulin; however, this was attenuated in the IHS group.

## 3. Discussion

Insulin receptor (Insr) resistance, due to various features associated with metabolic syndrome [19,20,21], leads to altered or impaired responses to circulating insulin. Insulin therapy is often very effective at treating the hyperglycemia associated with the resistance; however, the effects of the supplemental insulin on blood pressure (BP) and the cardiovascular-renal system in already hyperinsulinemic subjects are incompletely understood. Insr agonists and analogues that may limit these side effects are undergoing study and development; however, none are currently approved for use in humans.

To assess cardiovascular and renal impacts of insulin administration on insulin-resistant mice, we infused male TALLYHO/Jng mice with insulin for two weeks. TALLYHO/Jng mice were developed by Jackson Laboratory by selectively inbreeding mice of the Theiler Original strain for hyperglycemia [22]. Several multiple quantitative trait loci have been identified in these mice that are linked to adiposity, hyperglycemia, insulin resistance, and type 2 diabetes [16].

Few studies have examined BP in the TH mice, as of date. As compared to C57Bl6 mice examined in a previous study [5], we found TH mice had modestly higher (10–15 mm Hg) basal BP perhaps similar to obese, insulin-resistant humans [23]. Heart rate (HR) and pulse pressure (a measure of vascular compliance) were also higher in the TH, as was the light-to-dark ratio of MAP, an indicator of “non-dipping”, a classic sign of cardiovascular and endothelial dysfunction [24]. Many of these features are observed in humans with metabolic syndrome [25].

Insulin infusion (for two weeks) did not have major effects on blood pressure or heart rate even when coupled to feeding HSD. Systolic BP was significantly higher in the insulin-infused mice during the early time period of infusion; however this arose primarily due to the fact that systolic BP levels tended to fall in vehicle-infused mice. We do not fully understand this response, but it may reflect recovery from the surgeries to implant the radiotelemetry transmitter and osmotic pumps. The failure of the systolic BP to similarly fall in the insulin-infused mice could reflect volume expansion or increased sympathetic drive, due to insulin. In support of increased sympathetic drive, heart rate was higher in insulin-infused mice as well. However, the mice appeared to adapt to the HSD fairly quickly, and the BP elevation was not sustained. This indicated that the boost in circulating insulin did not dramatically increase salt-sensitivity of BP in these rodents. In humans, salt sensitivity of BP has been closely tied to insulin resistance of major target tissues. Some studies show the kidney does not develop resistance to the anti-natriuretic actions of insulin, thus sodium retention ensues [17]. In our study, we observed weight gain in both groups of insulin-infused mice. Whether this weight constituted adipose gain or partially extracellular fluid volume (ECFV) expansion is uncertain. The fact that hematocrit did not fall suggests that ECFV was fairly stable. Furthermore, if ECFV expanded, it did not raise BP, at least in the two week period of study.

One protective mechanism which may normalize BP in the face of insulin-induced sodium retention is the production of the vasodilator and natriuretic molecule, nitric oxide (NO) [26]. Insufficiencies in endothelial or renal epithelial tubular production of this molecule have been proposed to link insulin resistance to hypertension [27]. Moreover, dietary deficiency of NOx predisposes mice to endothelial dysfunction and early death [28]. A recently published study by Park et al. [29] found exogenous insulin provided to apolipoprotein E knockout mice reduced atherosclerosis, and the investigators postulated a role for improved endothelial function. Endothelial NOS (expressed in both the endothelium and epithelium of kidney) has been shown to be regulated by phosphorylation on several residues, including: Y81, S114, T495, S615, S633, Y657, and S1177 (human sequence) [30]. We chose to assess phosphorylation on two of these sites (S1177 and Y657) by Western blotting of whole tissue homogenates, as there was prior evidence to show insulin regulation of these sites [13,14].

Insulin infusion resulted in a number of changes in the kidney, per se, consistent with increased eNOS activity, including increased cortical NOS activity and reduced uncoupled (inactive) eNOS band density, as well as, increased whole-cell levels of eNOS in outer medulla and a borderline increase (*p* = 0.051) in cortex. P-S1177-eNOS phosphorylation was also increased in the outer medulla with a tendency for a similar increase in cortex (*p* = 0.069). Thus, these insulin-resistant rodents were not entirely resistant to molecular changes in eNOS that have been described previously in insulin-sensitive mice.

However, this activation was not reflected in increased circulating plasma concentrations or urine excretion of the major metabolic breakdown products of NO, i.e., nitrates and nitrites (NOx). In fact, these levels were significantly reduced by the end of the 2-week insulin infusion period. Thus, systemic NOx was reduced by chronic insulin infusion in these mice, a finding we did not observe in insulin-sensitive C57Bl6 mice [5]. For example, we’ve reported short-term (2–4 h after i.p. injection), insulin administration will increase urine NOx in C57Bl6 mice [6].

It is important to consider the role of nNOS (neuronal, NOS1) and iNOS (inducible, NOS2), the other two described isoforms of NOS, which may reflect on overall NOS activity measures and urine and plasma NOx levels. For example, neuronal NOS (nNOS or NOS1) can affect tubuloglomerular feedback, as it is heavily expressed in the macula densa [31]. We previously reported reduced protein levels of nNOS in the kidney in renal tubular Insr knockout mice (Insr^fl/fl^ Ksp-Cre) [7]. Recently insulin therapy was shown to reduce renal mitochondrial iNOS expression, as well as, NO, in a rat model of septic shock [32]. The treatment, overall, reduced oxidative stress and attenuated renal damage and loss of function. Thus, insulin may actively regulate all 3 isoforms of NOS in kidney, and many of these influences are beneficial.

We measured arginase activity in plasma and kidney in order to probe the inconsistencies between molecular changes observed in the kidney for eNOS versus excretion/circulation of NOx. Arginase, the biomanganese enzyme that converts L-arginine to ornithine, could potentially reduce substrate supply to NOS, thus provide a potential explanation for the fall in plasma and urine NOx with infusion [33]. However, we did not find an increase in the activity of this enzyme in either plasma or kidney tissue. Thus elevated arginase activity did not appear to be the root cause of this reduction in urinary or plasma levels of NOx. There is the potential for vascular NO production to be reduced, which may provide one explanation. Another possibility is that substrate, i.e., arginine levels are reduced by chronic insulin infusion. Thus, we must conclude that there is the possibility that BP would begin to rise if the study had been extended beyond this period, due to this fall in NOx.

Finally, insulin infusion resulted in alterations in blood chemistry that could be of concern. The marked fall in BUN may indicate hyperfiltration, or on the other hand, reflect the reduced intake of rodent chow (due to greater consumption of calories from sugar-sweetened water). Additional studies would be needed to clarify this finding. The increased anion gap suggests that insulin infusion plus sugar-sweetened drinking water causes mild acidosis. A high anion gap has been associated with insulin resistance, lower cardiorespiratory fitness, early kidney disease, and mortality in humans [34].

A potential confounder of our interpretation of the effects of hyperinsulinemia was the administration of sugar-sweetened drinking water to insulin-infused mice; however, in the absence of this dietary change, mice develop relative hypoglycemia, which would have its own set of confounds. We could not offer the control mice sugar-sweetened water as this would raise endogenous insulin levels. Thus, we chose to stay with the model we had developed in the C57Bl6 mouse study.

Taken together, our findings support the concept that that insulin infusion (in the semi-chronic time frame) in insulin-resistant rodents does not majorly affect BP and may even have some positive effects, such as increasing renal eNOS activity by increasing capacity (expression) and post-translational modifications, i.e., activating phosphorylation. However, the trend for increasingly lower urinary NOx, lower plasma NOx, and higher anion gap would suggest exacerbation of existing insulin resistance that might, over time, lead to worsening prognosis.

## 4. Materials and Methods

### 4.1. Animal Protocols

All animal studies were approved (protocol 16-011, approved February 10, 2016) by the Georgetown University Institutional Animal Care and Use Committee (IACUC) and conducted in accordance with the National Institutes of Health Guide for the Care and Use of Laboratory Animals. Insulin-resistant, TallyHo/Jng (TH, The Jackson Laboratory, Bar Harbor, ME, USA) male mice were studied between the ages of 4–7 months. Mice were bred at Georgetown University. Mice were randomly divided into 3 treatments: (1) control- received 0.9% saline infusion as vehicle; (2) insulin infused- received insulin (50 U/kg·bw/d); and (3) insulin-infused plus switched to high-NaCl diet (Envigo, TD.110078, Hungtington, UK) after 7 days. All mice were implanted with osmotic minipumps (Alzet, model 1002, Cupertino, CA, USA) and infused for 14 days. All mice received Purina Chow 5001, 1% NaCl diet for the first 7 days. Insulin-infused mice received ad libitum access to drinking water containing 20% glucose, 0.2% NaCl, and 0.2% K_2_HPO_4_ to help attenuate hypoglycemia and replace electrolytes (lost from consuming lower amounts of dry chow). Blood pressure (BP) was recorded by radiotelemetry (Dataquest ART™, version 4.36, Data Sciences Incorporated, St. Paul, MN, USA). Radiotelemetry transmitters were implanted in the carotid artery into mice 5 days prior to osmotic pumps and baseline BP and heart rate (HR) were recorded for 48 h prior to implanting pumps. Urine (24-h) was collected in additional mice (non-telemeterized) in mouse metabolic cages (Hatteras Instruments Incorporated, Cary, NC, USA) in a baseline period (untreated), and during days 1, 7, and 14 of insulin infusion or control. Kidneys and blood were collected on all mice under inactin anesthesia after 14 days of insulin infusion (or control conditions) plus/minus HSD.

### 4.2. Radiotelemetry

Mice in which BP was recorded were singly housed in conventional cages located on top of radio-receivers. Recordings were taken every 10 min for 10 s for the entire experiment as described elsewhere [35], except during a 4-h period when osmotic minipumps were implanted. Data was downloaded to Microsoft Excel spreadsheets for further analysis. Daily averages for mean arterial blood pressure (MAP) and HR were calculated for 24 h periods (12:00 a.m.–11:59 p.m.), as well as, 12-h (diurnal, day/night) periods. Lights were turned on at 6 a.m. and off at 6 p.m. in the room.

### 4.3. Blood, Plasma, and Urine Analysis

Heparinized blood collected at euthanasia was analyzed for chemistry using an iSTAT portable analyzer (Abbott, Chicago, IL, USA). Plasma was analyzed for insulin by ELISA (Crystal Chem) and nitrate and nitrites (NOx) by a colorimetric assay (Cayman Chemical Company, Ann Arbor, MI, USA). Volumes were recorded on urine (24-h) collected in metabolic cages and analyzed for NOx and creatinine (Cayman Chemical Company).

### 4.4. Western Blotting

Western blotting was performed on kidney regions. Briefly the left kidney was dissected into cortex, inner stripe of outer medulla (ISOM), and inner medulla (IM). Each region was homogenized separately in isolation solution (200 mM sucrose, triethanolamine buffer, pH = 7.5) using a bead homogenizer (Beadblaster™ 24, Benchmark, Sayreville, NJ, USA). Protein concentrations of the homogenates were determined by a bicinchionic acid assay (Pierce BCA Protein Assay, ThermoFisher Scientific, Waltham, MA, USA). Homogenates were solubilized in Laemmli sample buffer containing 30 mg/mL dithiothrietol (DTT). Coomassie-stained loading gels were performed prior to Western blotting to determine accuracy of protein determinations and quality of samples. Gels were loaded with 10–20 μg protein in each lane for blotting. For non-reducing conditions, cortex homogenates were solubilized in Laemmli buffer without the addition of DTT. Electrophoresis was performed with electrophoresis chambers packed with ice to reduce uncoupling of active eNOS protein complexes. For all conditions, blotting was performed on pre-cast mini gels (Criterion, TGX BioRad, Hercules, CA, USA) as described [35]. Chemiluminescence was used to visualize bands. β-actin band density was used to normalize for protein loading. Antibodies used included: β-actin (monoclonal mouse, Sigma-Aldrich, St. Louis, MO, USA), eNOS (NOS3 (monoclonal rabbit D9A5L, Cell Signaling, Danvers, MA, USA) phosphorylated (serine 1177)-eNOS (monoclonal rabbit C9C3, Cell Signaling), and phosphorylated (Tyrosine 657)-eNOS (NP4031, ECM Biosciences, Versailles, KY, USA).

### 4.5. Nitric Oxide Synthase (NOS) and Arginase Activity

Nitric oxide synthase (NOS) and arginase activities were assessed using colorimetric kits. The right kidney was frozen and stored (−80 °C) initially then thawed and dissected into the 3 kidney regions. Each region was homogenized in a proprietary buffer after adding cofactors and substrate so they were not-limiting (Biovision NOS activity kit, Milpitas, CA, USA). Arginase activity was assayed using the Arginase Activity Assay Kit (Sigma-Aldrich), which detected urea produced with unlimited addition of arginine and cofactors.

### 4.6. Statistics

BP and HR data were analyzed by two-way repeated measures analysis of variance (Time X Treatment, Graphpad Prism 7) to determine significant differences, as well as, interactions. One-way ANOVA (followed by a multiple comparison’s test when *p* < 0.05) was used to determine differences between groups when comparing Control (non-infused), insulin-infused (I), and insulin-infused plus high-NaCl diet (IHS) groups to each other.

## Figures and Tables

**Table 1:** Levels of blood/plasma components in control, insulin-infused and insulin-infused plus high-sodium diet (HSD) mice.

Blood Chemistry ^¥^				
Parameter	Control	Insulin	Insulin + HSD	One-Way ANOVA *p*-Value
Insulin (nM)	0.66 ± 0.23	2.37 ± 0.54	2.58 ± 0.56	0.015
Na^+^ (mM)	144 ± 0.7	149 ± 1.9 *	146 ± 0.6	0.030
K^+^ (mM)	4.6 ± 0.1	4.6 ± 0.2	4.4 ± 0.2	0.64
Cl^−^ (mM)	116 ± 0.9	115 ± 0.8	112 ± 0.7 ^†^	0.012
TCO2 (mM)	31.8 ± 0.9	27.8 ± 1.8	31.6 ± 0.8	0.058
BUN (mg/dL)	21.3 ± 0.7	6.9 ± 0.8 *	7.4 ± 0.7 *	<0.0001
Glucose (mg/dL)	201 ± 6	254 ± 36	226 ± 24	0.33
Hct (%)	31.9 ± 0.7	32.9 ± 0.8	33.9 ± 0.9	0.10
pH(−log_10_ [H^+^])	7.24 ± 0.02	7.11 ± 0.05 *	7.19 ± 0.01	0.027
HCO_3_^−^ (mM)	29.6 ± 0.9	25.4 ± 2.2	29.4 ± 0.9	0.10
Beecf (mM)	2.16 ± 1.24	−4.10 ± 3.12	1.18 ± 1.08	0.066
AnGap (mM)	3.25 ± 1.73	14.0± 3.8 *	8.64 ± 1.15	0.02
Hb (g/dL)	10.8 ± 0.2	11.2 ± 0.2	11.5 ± 0.3	0.20

^¥^ mean ± sem; * significantly different from control; ^†^ significantly different from insulin by one-way ANOVA followed by multiple comparisons testing; *n* = 10–12/treatment.
